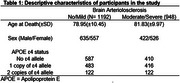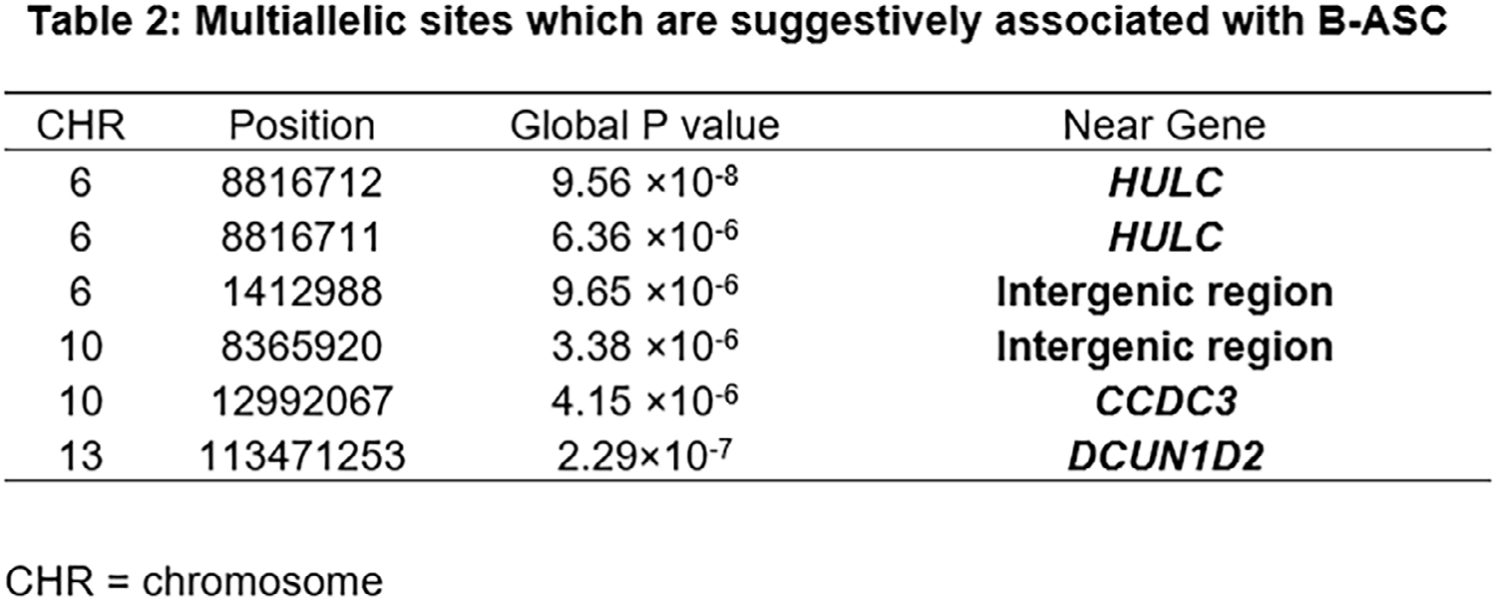# Unveiling the overlooked: Multiallelic Variants in Brain Arteriolosclerosis

**DOI:** 10.1002/alz70855_106206

**Published:** 2025-12-24

**Authors:** Khine Zin Aung, Xian Wu, Inori Tsuchiya, Lincoln MP Shade, Erin L. Abner, Peter T Nelson, David W. Fardo, Yuriko Katsumata

**Affiliations:** ^1^ Sanders‐Brown Center on Aging, University of Kentucky, Lexington, KY, USA; ^2^ Department of Biostatistics, College of Public Health, University of Kentucky, Lexington, KY, USA; ^3^ Department of Epidemiology and Environmental Health, University of Kentucky, Lexington, KY, USA; ^4^ Department of Pathology, University of Kentucky, Lexington, KY, USA

## Abstract

**Background:**

Brain arteriolosclerosis (B‐ASC), a subtype of small vessel pathology, is present in more than 50% of individuals over the age of 80 years and is associated with cognitive impairment, motor dysfunction, and sleep disturbance. We previously conducted an autopsy‐based genome‐wide association study (GWAS) and identified the B‐ASC‐associated single nucleotide polymorphisms (SNPs). To characterize the genetic architecture of B‐ASC, however, we need to investigate beyond SNPs. **Multiallelic variants** are likely to be ignored in GWAS because standard statistical analysis methods are designed for biallelic variants. In this study, we applied score‐based testing within the generalized linear model framework and explored multiallelic variant associations with autopsy‐confirmed B‐ASC in autosomal chromosomes.

**Method:**

We used whole‐genome sequencing (WGS) data from the Alzheimer's Disease Sequencing Project (ADSP) and B‐ASC phenotype data from the National Alzheimer's Coordinating Center (NACC) neuropathology (NP) dataset (September 2023 data freeze). We dichotomized the B‐ASC data (NACCARTE) into 0 = no/mild (*n* =  1,192) and 1 = moderate/severe (*n* = 948) (Table 1). The model included sex, age at death, and the top three principal components as covariates. We then computed global scores and the corresponding *p*‐values.

**Result:**

After variant filtering and quality control, 1,388,681multiallelic variants were retained for study. The genomic regions and genes listed in Table 2 (with their *p*‐values of less than 1×10^‐5^) indicate potential involvement of these loci in BASC. Further investigation of the identified genes (HULC, CCDC3, DCUN1D2) and associated intergenic regions may provide insights into the genetic basis of B‐ASC.

**Conclusion:**

We investigated possible associations between human multiallelic variants and B‐ASC risk. These are underexplored areas — both the genetic phenomena of multiallelic variants, and B‐ASC as a dementia‐driving pathology. Identifying novel genetic variants putatively contributing to the pathogenesis of B‐ASC will move the field forward although validation with independent datasets is required.